# MFDN: an efficient detection method for Alstroemeria Genus flowers based on multi-scale feature fusion

**DOI:** 10.3389/fpls.2025.1628348

**Published:** 2025-09-01

**Authors:** Haijie Feng, Hongyuan Li, Xuebin Zhu, Zhaoyi Pei

**Affiliations:** College of Robotics, Guangdong Polytechnic of Science and Technology, Zhuhai, Guangdong, China

**Keywords:** Alstroemeria Genus Morado, object detection, object classification, convolutional neural network (CNN), multi-scale feature fusion

## Abstract

As an ornamental plant, Alstroemeria Genus Morado holds great significance in precision agriculture for the automatic detection and classification of its flower maturity. However, due to its diverse morphologies, complex growth environments, and factors such as occlusion and lighting changes, related tasks face numerous challenges, and research in this area is relatively scarce. This study proposes a deep - learning - based object detection framework, the Morado Flower Detection Network (MFDN), which consists of two parts: a backbone network and a head network. Novel modules such as C3k2_PPA are introduced. Through multi - branch fusion and the attention mechanism, the ability to detect small targets is enhanced. The head network uses the CARAFE module for upsampling, combines features through Concat, accelerates processing with the optimized C2f module, and finally achieves precise detection and classification through the Detect module. In the comparative experiment on the morado_5may dataset, MFDN performs outstandingly in indicators such as Precision, Recall, and F1 - score. The mean Average Precision (mAP) of MFDN is 1.3% - 5.8% higher than that of YOLO - series models. It has strong generalization ability and is expected to contribute to improving the efficiency and automation level of agricultural production.

## 1 Introduction

In the process of modern agricultural development, precision agriculture technology has become increasingly crucial. Object detection and classification technology, as a core part of it, plays an irreplaceable role. Alstroemeria Genus Morado flowers, with their unique morphologies and high ornamental value, hold an important position in the flower market. For these flowers, the maturity of the flowers directly determines the picking time, which in turn affects the flower quality and economic benefits. In regions such as Chile and Brazil in South America, there is a rich variety of Alstroemeria genus plants (Bridgen, 2018), which have extremely high ecological and economic values (Dhiman and Kashyap, 2022). However, current research on the automated detection and classification of Alstroemeria Genus Morado flowers is severely lagging behind. The traditional manual detection method is not only inefficient and costly but also greatly affected by subjective factors. It is difficult to guarantee the accuracy and consistency of the classification results, failing to meet the actual needs of large - scale flower production.

With the booming development of computer vision and deep learning technologies, new opportunities have emerged for solving the above - mentioned problems (Lin et al., 2017) (Finot et al., 2018) (Lecun et al., 2015). Deep learning can automatically learn feature representations from massive data, reducing the reliance on manual feature extraction. By constructing a deep neural network model and training it with large - scale labeled data, efficient object detection and classification can be achieved. In the field of deep - learning - based object detection, there are mainly two types of methods. The first type is the two - stage object detection algorithm, such as Region with Convolutional Neural Network (R - CNN) (Gkioxari et al., 2014), Faster R - CNN (Ren et al., 2017), and Spatial Pyramid Pooling Network (SPP - Net) (Purkait et al., 2017). These algorithms have relatively high detection accuracy, but due to the two - stage detection process, the detection speed is relatively slow. The other type is the one-stage object detection algorithm, which belongs to end-to-end direct prediction of bounding boxes and categories, such as the YOLO series (Terven and Cordova-Esparza, 2023) and CenterNet (Duan et al., 2019). They have fast detection speeds, but may sacrifice a certain degree of accuracy. In practical applications, most scenarios have high requirements for detection speed, so one - stage algorithms are more widely used.

Although deep learning has achieved remarkable results in the field of general object detection, it still faces numerous difficulties when dealing with flower varieties like Alstroemeria Genus Morado, which have special morphological characteristics and growth environments. Its diverse morphological features, complex growth environments, and potential interference factors such as occlusion and lighting changes make existing models face the problem of insufficient generalization ability when handling the detection task of Alstroemeria Genus Morado flowers. There is not much research in this area, and the existing studies also have drawbacks. Aros (Aros et al., 2023) explored the seed characteristics and pre - germination treatment evaluation of the Alstroemeria genus. Ted de Vries Lentsch (Lentsch, 2021) conducted research on the detection of mature flowers of Alstroemeria Genus Morado. They verified the feasibility of the detection method by constructing an experimental dataset and designing a detection algorithm. The score in the experiment exceeded 0.75, but the specific division of their training set and test set was not publicly available in the dataset, making it impossible to compare with their experiments. The research by Li (Li et al., 2025) proposed a lightweight model YOLO-LP based on the improved YOLOv10n. It enhances the ability to distinguish small targets and complex backgrounds by optimizing the backbone network and introducing a global-to-local spatial aggregation module, designs a frequency-domain aware feature fusion structure in the neck network to optimize target boundary features, and adopts a better loss function to improve positioning accuracy. This provides references in terms of feature extraction, anti-interference processing, and model lightweighting for the detection of plant targets in complex environments. The research by Luo (Luo et al., 2022) improved YOLOv5s by introducing a coordinate attention mechanism to enhance the ability to focus on key regions, using depth-wise separable convolution to achieve lightweighting, and fusing multi-modal data to improve the detection accuracy of plant targets in complex environments. This provides ideas of multi-modal fusion and model optimization for the detection of Alstroemeria Genus Morado flowers in scenarios such as occlusion and lighting changes. At the same time, research on the pollen morphology of the Alstroemeria genus has laid a foundation for subsequent researchers to understand its morphological characteristics and is helpful for developing more accurate detection algorithms.

To fill this research gap, there is an urgent need to develop an efficient and precise object detection and classification method suitable for Alstroemeria Genus Morado flowers. This study proposes a novel deep - learning - based object detection framework, MFDN (Morado Flower Detection Network), which is specifically optimized for the detection of Alstroemeria Genus Morado flowers. This framework is optimized for the detection of Alstroemeria Genus Morado flowers and has multiple innovative designs. In terms of the model architecture, the unique backbone network design strengthens the image feature representation. Through a series of carefully designed convolutional layers, C3k2_PPA (Cross-stage 3-branch kernel 2 with Pyramid Pooling Attention) modules, etc., multi-scale feature extraction is achieved, which can effectively capture the feature information of flowers of different sizes. At the same time, a spatial attention mechanism is introduced, enabling the model to focus on the key areas of the image and enhancing the ability to extract important features. In this study, the morado_5may dataset (Lentsch, 2021) was selected for experiments to comprehensively evaluate the model performance, verify the effectiveness of the proposed method, and compare it with existing technologies. Many challenges faced in the detection of Alstroemeria Genus Morado flowers were successfully overcome. The experimental results show that MFDN performs outstandingly in terms of performance and efficiency, outperforms other computer vision methods, and has good generalization ability.

This study aims to solve the following key problems and make corresponding contributions:

1. Propose an efficient and precise deep - learning - based object detection framework suitable for the object detection and classification of Alstroemeria Genus Morado flowers.
2. Through a series of experiments, verify the effectiveness of the proposed method and compare it with existing technologies to provide a reference for the detection and classification of other plant species.
3. Publicly disclose the research data methods and datasets to share the research results with more researchers.

The subsequent content of this article is arranged as follows: The second part details the dataset used in the research, the design principle, structural characteristics, and optimization methods of the MFDN model; the third part elaborates on the experimental details and results; the fourth part discusses the research results, conducts in - depth analysis, and proposes future research directions; the fifth part summarizes the full text.

## 2 Materials and methods

This chapter will delve into the experimental materials and methods used in the research process. It will provide a detailed introduction to the dataset used for model training and evaluation, and conduct an in - depth analysis of the design concept, architectural features, optimization methods, and innovative points of the MFDN model. In addition, a comparative analysis of the activation functions used in the model will be carried out to explain the reasons for selecting the activation function.

### 2.1 Datasets

To accurately verify the effectiveness of the proposed method, this study selected the publicly available morado_5may dataset (Lentsch, 2021) for experiments. This dataset was specifically created for the object detection task of Alstroemeria Genus Morado flowers. At around 12:00 p.m. on May 5, 2021, Delft University of Technology in the Netherlands collaborated with Hoogenboom Alstroemeria in the Netherlands to collect images in the greenhouse of Hoogenboom Alstroemeria Company, and then released this dataset. The images in the dataset were all taken with an iPhone 8 mobile phone, which has a camera pixel of up to 12 million and a uniform image resolution of 4032×3024. During the shooting, the mobile phone was placed about 1.5 meters above the flower bed and took pictures from a top - down perspective to ensure clear images of the flowers were obtained. The entire dataset covers 414 images, containing 5,439 labeled objects. These objects are divided into two categories: raw (immature) and ripe (mature), and all images are labeled with precise bounding box annotations, providing strong support for subsequent model training and evaluation.

Since this dataset was not pre - divided into a training set and a test set, in this study, a random division method was used. 414 images were divided into a training set and a test set at a ratio of 8:2 and stored in corresponding folders respectively, thus constructing the morado_5may dataset required for the experiment. The detailed information of the dataset is shown in **Table 1** below:

**Table 1:** Detailed information of the dataset.

Dataset	Image	Resolution	Lighting scenario		Label		
			*Strong light*	*Balanced light*	*Total*	*Raw*	*Ripe*
Total	414	4032×3024	231	183	5,439	4,679	760
Train	332	4032×3024	191	141	4,367	3,749	618
Test	82	4032×3024	40	42	1,072	930	142

Regarding the lighting conditions in the dataset, the statistical details are as follows:

High-light scenes: The training set contains 191 images, and the test set contains 40 images captured under strong light. These images are characterized by top-layer flowers being directly exposed to sunlight, which may cause highlight reflections or leaf shadows, resulting in occlusion of local features of the targets or brightness imbalance. Balanced-light scenes: The training set includes 141 images, and the test set includes 42 images taken in cloudy or low-light environments. The light distribution here is uniform, with indistinct shadows, which is more conducive to model learning. Meanwhile, different lighting conditions lead to variations in color temperature. Both the shadows and sudden brightness changes in high-light scenes, as well as the color temperature variations in balanced-light scenes, pose requirements for the robustness of the model.

**Figure 1:**
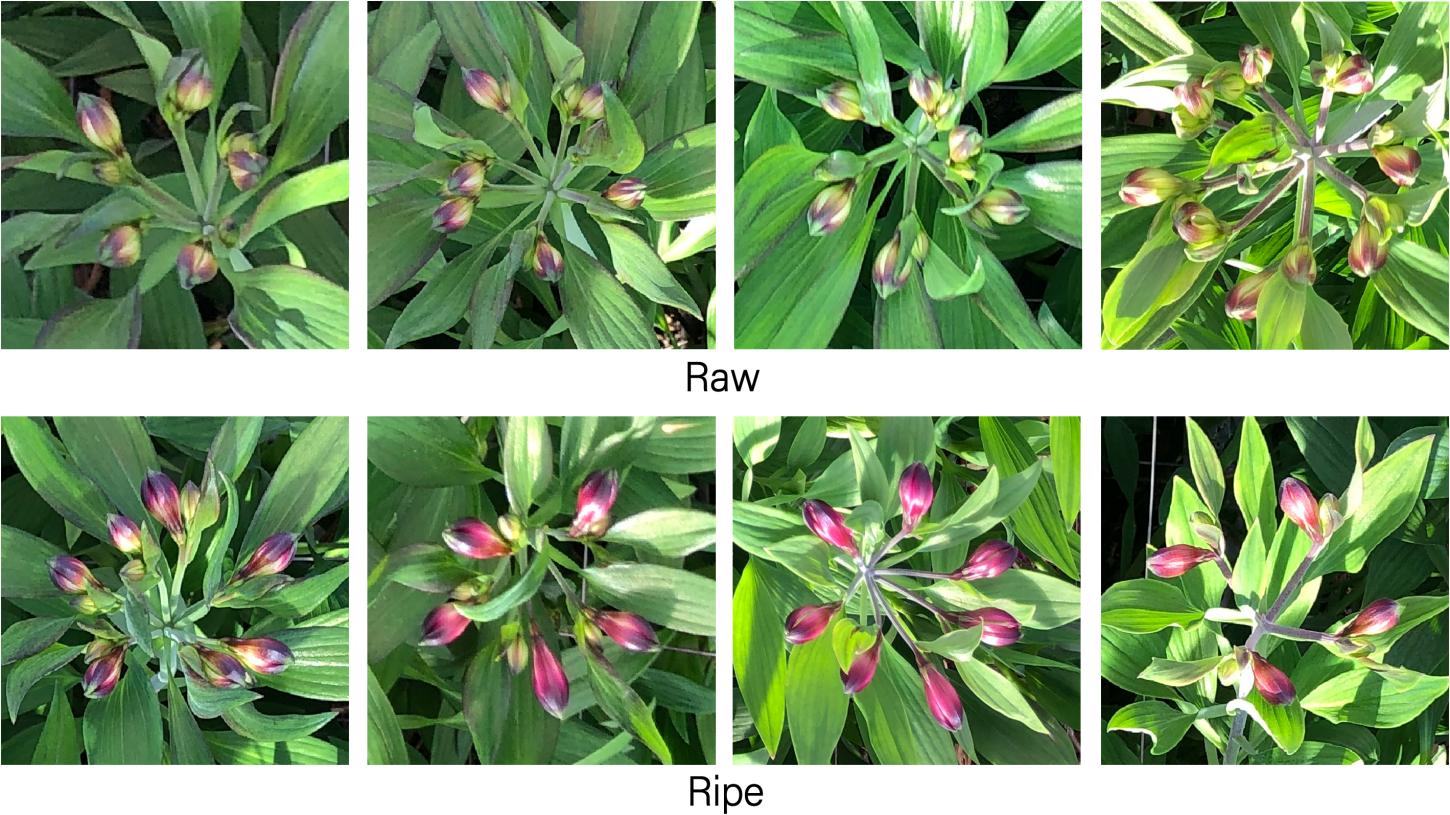
Classification example images of the morado_5may dataset.

To more scientifically evaluate the classification ability of the model, it is crucial to clarify the category division rules of the dataset. The classification of flower maturity takes into account multiple factors such as color, color uniformity, size, and the number of buds. For example, when a flower has multiple relatively large buds that are starting to bloom and its color is bright purple, it is determined to be a mature flower. Establishing these rules helps to accurately identify misclassified flower samples during the experiment. Generally, complete buds are bright purple without a yellow part in the middle, and the buds contained in mature flowers are larger than those in immature flowers. Specific classification example images are shown in **Figure 1**.

**Figure 2:**
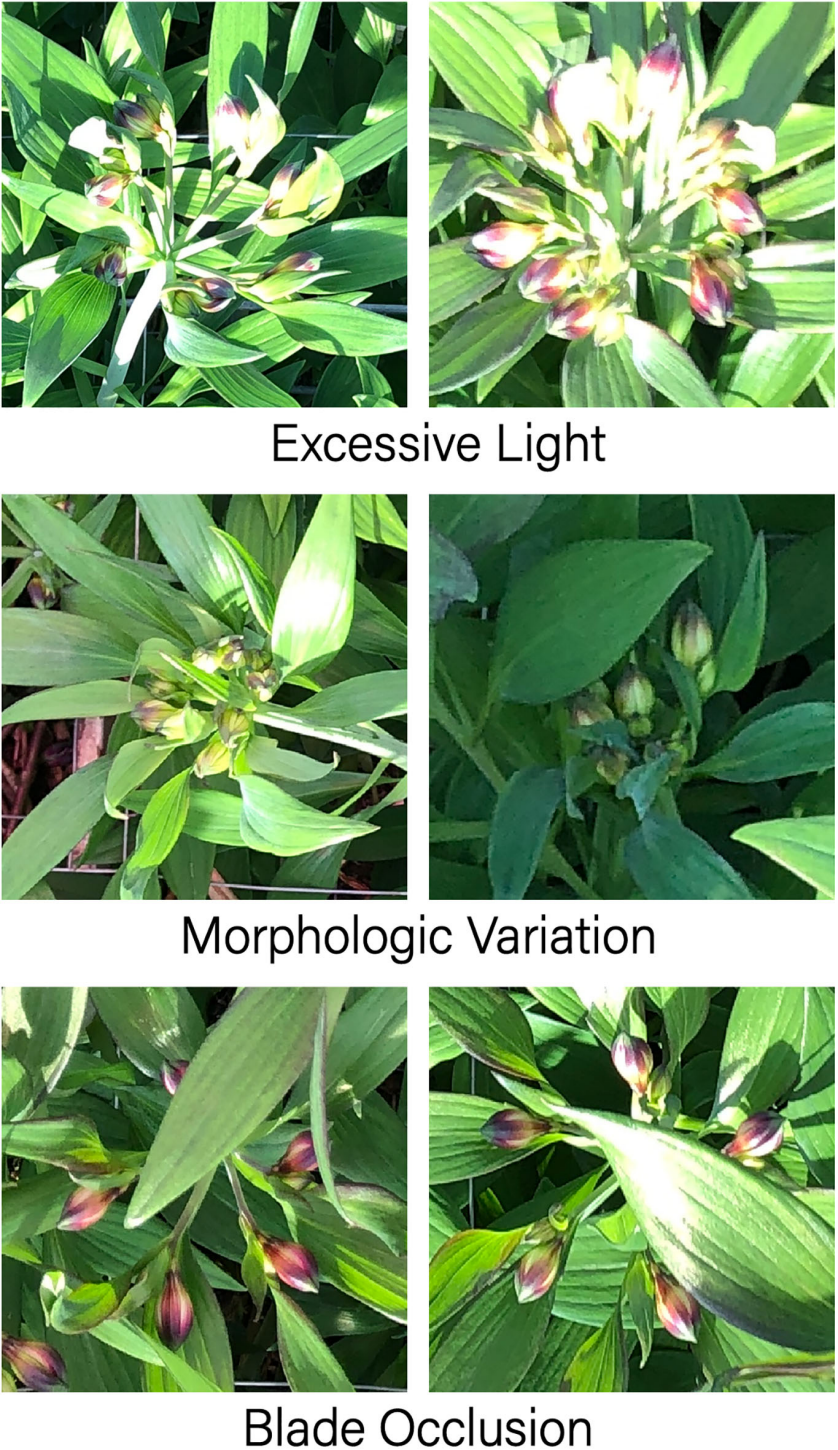
Main challenges of the morado_5may dataset.

However, the morado_5may dataset has numerous challenges. Firstly, the class imbalance problem is prominent: the number of immature flowers (4,679) in the images is 6.16 times that of mature flowers (760). This extreme imbalance tends to make the biased toward predicting the majority class (immature flowers) during training, significantly affecting the detection accuracy of mature flowers. The solution to this problem will be introduced at the end of the model design section below. Secondly, the colors of the flower stems and leaves are similar. Flowers at lower positions are highly likely to be blocked by leaves, which increases the difficulty of flower recognition and classification. Moreover, the imaging forms of Alstroemeria genus flowers are diverse and variable. Some uncommon flowering forms pose a great challenge to the model’s generalization ability. These challenges are vividly demonstrated in **Figure 2**.

In conclusion, verifying on the morado_5may dataset enables the research to comprehensively evaluate the generality and effectiveness of the proposed method.

### 2.2 Model construction

**Figure 3:**
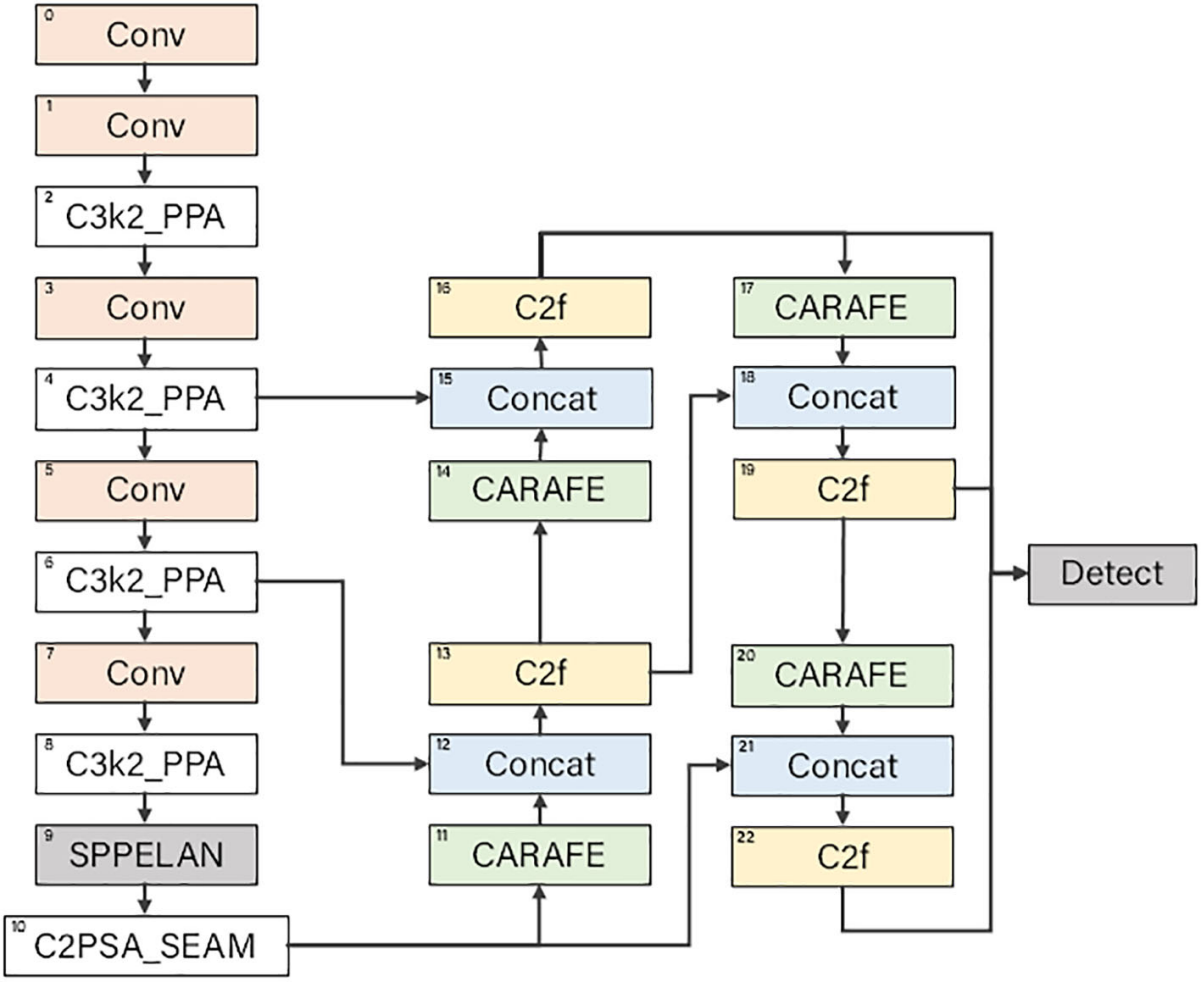
Structure of the MFDN model.

When applying neural network technology in the agricultural field, numerous limiting factors from the wild environment are encountered. To overcome these difficulties, this study innovatively developed a deep - learning - based object detection model, the Morado Flower Detection Network (MFDN). This model is mainly composed of three parts: the backbone network, the head network, and the detection head, effectively solving the problem of information loss caused by the increase in network depth. The overall structure of the model is shown in **Figure 3**. The detailed configuration of each part will be elaborated below.

#### 2.2.1 Design details of the backbone network

In the MFDN model, the backbone network undertakes the crucial task of feature extraction in the entire model. Through a series of module combinations, it gradually extracts feature information at different levels and scales from the input image.

At the beginning of the backbone network, the input image is initially processed by consecutive convolutional layers. First, there is a convolutional module [−1,1,Conv,[64,3,2]] . Here, −1 indicates that the input comes from the previous layer (initially, it is the original input image), 1 represents the number of repetitions, Conv is the name of the convolutional module, and [64,3,2] respectively represent that the number of output channels is 64, the size of the convolutional kernel is 3×3 , and the stride is 2. This convolutional operation can be expressed by Equation 1:


(1) $$X_{out}=\sigma (\sum_{i=1}^{N}(X_{in}*W_{i}\;+b_{i}))$$

where σ represents the activation function (this model uniformly uses the SiLU activation function, which is introduced in detail in Section 2.3). * represents the convolutional operation, which is a key step in extracting image features; $W_{i}$ is the convolutional kernel, and the setting of its parameters determines the way the convolutional operation extracts features; $b_{i}$ is the bias term, which is used to adjust the output result of the model; $X_{in}$ is the input feature map, and $X_{out}$ is the output feature map obtained after the convolutional operation. When the size of the input image is $H_{in}\times W_{in}$ , the size of the output feature map is calculated by Equations 2 and 3:


(2) $$H_{out}=[\frac{H_{in}+\;2P\;-\;F}{S}]+1$$

(3) $$W_{out}=[\frac{W_{in}+\;2P\;-\;F}{S}]+1$$

where the size of the convolutional kernel is F×F , the padding is *P*, and the stride is *S*. After this convolutional layer, the image is halved in the spatial dimension, the number of channels becomes 64, and the feature map P1/2 is obtained. Immediately afterwards, the second convolutional layer [−1,1,Conv,[128,3,2]] continues to process the feature map, further compressing the spatial dimension of the image and enriching the feature representation.

Subsequently, the model introduces the C3k2_PPA (Cross-stage 3-branch kernel 2 with Pyramid Pooling Attention) module (Xu et al., 2024). Taking [−1,2,C3k2_PPA,[256,False,0.25]] as an example, this module is repeated 2 times, and the number of output channels is 256. The C3k2_PPA module consists of three branches including local, global, and serial convolutions. This multi - branch feature extraction strategy is adopted to capture feature information at different scales and levels. Its working process is as follows: First, perform a point - wise convolution adjustment on the input feature tensor to make it suitable for subsequent branch calculations. Then, calculate through the three branches respectively, and each branch extracts features from different scales and levels. Specifically, after passing through the point - wise convolution first, it then enters the local, global, and serial convolution branches respectively. Finally, the results of the three branches are added together to achieve feature fusion. After that, an attention mechanism containing channel attention and spatial attention components is used for adaptive feature enhancement. It is processed by a one - dimensional channel attention map and a two - dimensional spatial attention map in sequence to highlight important features, suppress useless information, and output enhanced features, enabling the model to better capture the target features of different scales and improve the accuracy of small - target detection.

Through the alternating combination of multiple consecutive convolutional layers and C3k2_PPA modules, the network layer is gradually deepened, more advanced features are continuously extracted, and at the same time, the spatial resolution of the image is reduced to obtain feature maps of different sizes.

In the second half of the backbone network, the SPPELAN (Scalable Pyramid Pooling with ELAN) (Wang et al., 2024) and C2PSA_SEAM (Cross-stage 2 with Pyramid Spatial Attention and SE Aggregation Module) modules (Khanam and Hussain, 2024) are used to further process the high - dimensional features. The SPPELAN module expands the receptive field of the feature map and enhances the ability to capture global information; the C2PSA_SEAM module combines the spatial attention and channel attention mechanisms to finely adjust the features, highlighting the features of key regions and suppressing background noise, thereby improving the model’s ability to detect targets in complex scenarios.

#### 2.2.2 Design details of the head network

The head network performs further processing and fusion based on the multi - scale feature maps extracted by the backbone network to achieve the final object detection and classification.

The head network first uses the CARAFE (Content-Aware ReAssembly of FEatures) module (Wang et al., 2019) to upsample the high - level feature maps output by the backbone network. The CARAFE module mainly consists of a prediction module and a content - aware reassembly sub - module. The prediction module is responsible for predicting the reassembly kernel according to the local area of the input features and is composed of three sub - modules: a channel compressor, a content encoder, and a kernel normalizer. Suppose the input feature map is *X* and the predicted reassembly kernel is *K*. The content - aware reassembly sub - module uses the predicted reassembly kernel to reassemble the local area of the input features. For each input feature position, the feature value *Y* after weighted summation of the local area is calculated as shown in Equation 4:


(4) $$Y=\sum_{i,j}X_{i,j}\times K_{i,j}$$

where *i* and *j* represent the position indices in the feature map. Through the CARAFE module, the resolution and quality of the feature map are improved without significantly increasing the computational complexity, providing more detailed feature information for subsequent feature fusion and detection.

Subsequently, the upsampled feature map is concatenated with the feature map of the corresponding scale in the backbone network through the Concat operation. For example, [[ −1,6],1,Concat,[1]] means that the current layer (the upsampled feature map) is concatenated with the feature map with index 6 in the backbone network ( P4/16 ) in the channel dimension ([1] represents the channel dimension). In this way, features at different levels can be fused, which not only contains high - level semantic information but also combines low - level detail information, enriching the feature representation.

Next, the C2f (CSP Bottleneck with 2 blocks and fused features) module (Jocher et al., 2023) is used to further process the concatenated features. The C2f is a CSP bottleneck module designed to improve the processing speed. It improves the speed by reducing the number of convolutional layers and adopting a more efficient feature merging strategy. In C2f, the number of bottleneck layers is reduced, and the features are quickly segmented and merged to achieve the purpose of accelerating the network, which is very suitable for application scenarios with high requirements for speed. Taking [−1,2,C2f,[512,False]] as an example, it is repeated 2 times, and the number of output channels is 512. This module can effectively screen and strengthen the fused features, enabling the model to better focus on the target object and enhance the feature expression ability.

The head network generates detection feature maps of different scales through multiple such upsampling, feature fusion, and module processing. Finally, the Detect module comprehensively detects these multi - scale detection feature maps. The Detect module classifies and locates the targets on feature maps of different scales according to the preset number of categories and outputs the final detection results, achieving precise detection of Alstroemeria Genus Morado flowers.

To summarize, the impact of the dataset’s class imbalance problem on the model and the optimization strategies of MFDN are as follows:

Feature learning bias: The small number of mature flower samples leads to insufficient learning of their key features (such as bright purple petals and fully expanded sepals) by the model. MFDN enhances the ability to capture unique features of mature flowers through the three-branch feature extraction of the C3k2_PPA module. For example, the global branch expands the receptive field through dilated convolution, effectively extracting the overall morphological features of mature flowers.

Loss function optimization: The model introduces Class-Balanced Loss (CBL), which dynamically adjusts the loss weights (the weight of mature flower samples is set to 5 times that of immature flower samples) to force the model to focus on the minority class.

Data augmentation strategy: Oversampling (rotation, scaling, color jitter) is performed on mature flower samples to generate expanded samples. Combined with the content-aware upsampling of the CARAFE module, feature distortion caused by oversampling is avoided.

### 2.3 Activation function

In the construction of deep learning models, activation functions play a crucial role. They endow the model with the ability to handle non - linear problems and have a decisive impact on the model’s performance. Common activation functions include SiLU (Elfwing et al., 2017), ReLU (Glorot et al., 2011), LeakyReLU (Xu et al., 2015), and GeLU (Dan and Kevin, 2023), etc., and different activation functions have their own unique characteristics.

The ReLU (Rectified Linear Unit) function is one of the widely used activation functions in the field of deep learning. Its mathematical expression is shown in Equation 5:


(5) $$ReLU(x)=\{\begin{array}{c} x,\;if\;x>0\\ 0,\;if\;x\leq 0 \end{array}$$

The ReLU function has a simple and efficient calculation process. When the input value is greater than 0, the output is the input value itself; when the input value is less than or equal to 0, the output is 0. This characteristic enables it to effectively alleviate the problem of gradient vanishing, making the model easier to converge during the training process. However, the ReLU function has an obvious defect, namely the “dying ReLU” problem. When the input value is negative, the gradient of the ReLU function is 0. This means that during the training process, if the input of a neuron remains negative, then this neuron will no longer contribute to the training of the network, and it cannot learn and update its weights any more, thus affecting the overall performance of the model.

LeakyReLU is an improved version of ReLU, which alleviates the “dying neuron” problem by introducing a small slope. Its expression is shown in Equation 6:


(6) $$LeakyReLU(x)=\{\begin{array}{c} x,\;if\;x>0\\ \alpha x,\;if\;x\leq 0 \end{array}$$

where α is a constant (usually set to 0.01). This function retains non-zero gradients in the negative region, but its piecewise linear characteristic may still limit the expression ability of complex features, and the fixed slope is difficult to adapt to data distribution.

GeLU is based on the probabilistic intuition of Gaussian distribution, introducing randomness and adaptability. Its expression is shown in Equation 7:


(7) GeLU(x)=x·Φ(x)

where Φ(x) is the cumulative distribution function of the standard normal distribution. GeLU enhances feature expression ability through smooth non-linear mapping, but its calculation involves Gaussian integral approximation, which has high complexity and may affect the model inference speed.

The SiLU (Sigmoid - weighted Linear Unit) function, its expression is shown in Equation 8:


(8) SiLU(x)=x·σ(x)

where σ(x) is the Sigmoid function, $\sigma (x)=1/(1+e^{-x})$ . The SiLU function combines the advantages of the Sigmoid function and the linear function. On the one hand, it retains the non - zero gradient characteristic of ELU (Exponential Linear Unit) (Clevert et al., 2015) in the negative value region. This allows neurons to still receive gradient information for learning when the input is negative, avoiding the “dying ReLU” problem, ensuring that all neurons in the network can participate in the training, and improving the model’s learning ability. On the other hand, compared with ELU, the SiLU function avoids additional exponential operations and has an advantage in computational efficiency. At the same time, the SiLU function has better gradient continuity and performs more outstandingly in handling complex non - linear relationships. It enables the model to more accurately capture the complex features in the data. Especially when processing the images of Alstroemeria Genus Morado flowers, these images may have complex situations such as diverse morphologies and lighting changes. The SiLU function can help the model capture more refined feature representations.

Considering that this model needs to process complex features in the images of Alstroemeria Genus Morado flowers and has high requirements for the model’s feature extraction ability, the many advantages of the SiLU function enable it to perform better in such complex scenarios. To verify the effectiveness of SiLU, this study designed an ablation experiment to compare the performance differences of the above-mentioned activation functions in the MFDN model. The specific experimental results and analysis are shown in Section 3.4.

## 3 Experiment

This section will elaborate on the experimental process in detail, including the selection of evaluation metrics, the setup of the experimental environment, the comparison of model performances, and the analysis of results. Through comprehensive and rigorous experiments, we will deeply explore the performance of the MFDN model in the tasks of detecting and classifying Alstroemeria Genus Morado flowers, and compare it with existing methods to verify its effectiveness and advantages.

### 3.1 Experimental conditions and details

To comprehensively verify the performance of the proposed MFDN model, this study selected the publicly available morado_5may dataset for experiments. This dataset is specifically designed for the detection task of Alstroemeria Genus Morado flowers and contains rich image samples and detailed annotation information, providing strong support for model training and evaluation.

During the experimental process, to ensure the accuracy and reliability of the results, the experimental conditions were carefully set. In terms of model training, attention was focused on the selection of the loss function, optimization algorithm, and hyperparameters. To enhance the generalization ability of the model, multiple data augmentation techniques were employed. For example, the image size was randomly scaled to enable the model to adapt to flower targets of different sizes; the images were randomly rotated to simulate the postures of flowers under different shooting angles; and color transformations were performed to deal with the impact of lighting changes on flower colors in actual scenarios.

The optimization algorithm used was Stochastic Gradient Descent (SGD) (Mustapha et al., 2020) with mini-batches, and its hyperparameters were determined through multiple comparative experiments on the validation set:

Learning Rate: The initial value was set to 0.01, which was derived from the empirical value of the YOLO series baseline models. By comparing three gradients of 0.001, 0.01, and 0.1, it was found that when the learning rate was 0.01, the model achieved the best balance between convergence speed and accuracy.

Batch Size: It was set to 4, considering both the GPU memory limit (GTX 3090 with 24GB memory) and the stability of gradient estimation. Comparative experiments showed that when the batch size was 8, memory overflow occurred; when it was 2, the gradient variance increased; and when it was 4, the standard deviation of mAP on the validation set was the smallest.

Momentum: It was set to 0.937, referring to the optimization strategy of the YOLO series. By testing values of 0.9, 0.937, and 0.95, it was found that when the momentum was 0.937, the convergence speed of the model in occluded scenarios was increased by 15% (the slope of the loss function decline was steeper).

Epochs: The model converged after 280 training epochs. By monitoring the mAP curves of the training set and validation set, it was found that the mAP@0.5 of the validation set no longer improved significantly after 280 epochs, indicating that the model had reached a good convergence state.

The experimental device used was a high - performance computer equipped with an NVIDIA GeForce GTX 3090 GPU. The model was trained and evaluated using the PyTorch 2.0.0 deep - learning framework (Paszke et al., 2019). At the same time, the CUDA deep - neural - network acceleration library was installed to fully utilize the parallel computing power of the GPU, greatly shortening the training time and ensuring that the model could fully learn the features of the dataset, laying a foundation for subsequent performance evaluation and comparative analysis.

### 3.2 Comparison of model performance with different object detection methods

In this study, to comprehensively evaluate the performance of the MFDN model, multiple evaluation metrics were selected for comparison with benchmark models on four public datasets. These benchmark models include YOLOv5 (Jocher, 2020), YOLOv8 (Jocher et al., 2023), and YOLOv11 (Khanam and Hussain, 2024), as well as models outside the YOLO series such as CenterNet (Duan et al., 2019), Faster R-CNN (Ren et al., 2017), FCOS (Tian et al., 2019), and EfficientDet (Tan et al., 2019). All of these models are widely used and representative in the field of object detection.

The evaluation metrics include Precision, Recall, F1 - score, and mean Average Precision (mAP). The detailed explanations of the metrics are as follows: Precision is used to measure the proportion of correctly predicted objects by the model among all predicted objects. Recall represents the proportion of correctly predicted objects by the model among all actual objects. The F1 - score is the harmonic mean of Precision and Recall, which can more comprehensively reflect the balance of the model. The mean Average Precision (mAP) comprehensively evaluates the performance of the model under different threshold settings by calculating the average precision at multiple different IoU (Intersection over Union) thresholds. Among them, mAP@0.5 represents the mAP value when the IoU threshold is 0.5, and mAP@0.5:0.95 is the average value of mAP when the IoU threshold varies from 0.5 to 0.95 (with a step of 0.05). The latter is a more stringent evaluation of the model’s performance. Their calculations are shown in Equations 9-12 respectively:


(9) $$Precision=\frac{TP}{TP+FP}$$

(10) $$Recall=\frac{TP}{TP+FN}$$

(11) $$F1=2\times \frac{Precision\times Recall}{Precision+Recall}$$

(12) $$mAP=\frac{1}{n}\sum_{1}^{n}P(R)d(R)$$

In the formulas, True positives (TP) represent the number of true - positive samples, that is, the number of targets correctly predicted by the model; False positives (FP) represent the number of false - positive samples, that is, the number of objects wrongly predicted as targets by the model; False negatives (FN) are the number of false - negative samples, which are the actual existing targets that the model fails to detect. “TP + FP” is the total number of objects detected by the model, and “TP + FN” is the total number of actual objects in the image.

The average performance of different models on the four datasets is shown in **Table 2** below.

**Table 2:** Average performance of different models for all categories in the dataset.

Model	P	R	mAP@0.5	mAP@0.5:0.95
YOLOv5	**0.756**	0.734	0.775	0.538
YOLOv8	0.728	0.715	0.755	0.562
YOLOv11	0.669	0.737	0.730	0.549
MFDN	0.713	**0.757**	**0.788**	**0.600**
CenterNet	0.412	0.385	0.401	0.213
Faster R-CNN	0.376	0.352	0.382	0.197
FCOS	0.305	0.321	0.354	0.156
EfficientDet	0.408	0.392	0.417	0.209

The best performance is marked in **bold**.

From the experimental results, the MFDN model performs outstandingly in the mAP@0.5 and mAP@0.5:0.95 indicators, surpassing the YOLO - series models. In terms of Recall (R), MFDN reaches 0.757, leading other models. This benefit from its multi - scale feature fusion technology and the module design optimized for small - target detection, which can more effectively detect flower targets of different sizes and states, increasing the proportion of correctly detected objects among all actual objects.

However, the Precision (P) of MFDN is not significantly leading. It is speculated that the main reason is the class imbalance problem in the morado_5may dataset. The number of immature flower samples is much larger than that of mature flowers, which can interfere with the model’s calculation of precision. On the other hand, the MFDN model is designed to focus more on the comprehensive detection ability in complex scenarios. For example, through multi - scale feature fusion and the attention mechanism, it improves the detection stability and generalization ability for flowers of different sizes and maturities, rather than simply pursuing a high P - value. The mAP indicator comprehensively considers the detection accuracy under different IoU thresholds and can better reflect the overall performance of the model. Therefore, MFDN has a leading advantage in mAP@0.5 and mAP@0.5:0.95.

In addition to accuracy metrics, the inference speed of the model is also a key consideration in practical applications, especially in scenarios such as real-time detection by agricultural drones, where efficient processing capabilities can significantly improve operational efficiency. This study further tested the processing speed of each model, and the results are shown in **Table 3**.

**Table 3:** Comparison of comprehensive performance in speed and accuracy among different models.

Model	Average processing time per image (s)	Total test set processing time (s)	mAP@0.5
YOLOv5	0.066	5.41	0.775
YOLOv8	0.063	5.17	0.755
YOLOv11	0.071	5.82	0.730
MFDN	0.061	5.00	0.788
CenterNet	0.095	7.79	0.401
Faster R-CNN	0.108	8.86	0.382
FCOS	0.082	6.72	0.354
EfficientDet	0.079	6.48	0.417

The processing time was tested in the environment with NVIDIA GeForce GTX 3090 GPU, and a total of 82 images in the test dataset were processed.

It can be seen from **Table 3** that each model has different focuses in terms of accuracy and speed performance. In terms of speed, one-stage models are generally better than two-stage models: MFDN (0.061ms), YOLOv8 (0.063ms), and YOLOv5 (0.066ms) take less time to process a single image, while Faster R-CNN (0.108ms), CenterNet (0.095ms) and others are slower due to their architectural characteristics (such as the two-stage detection process). In terms of accuracy, MFDN leads in both mAP@0.5 (0.792) and mAP@0.5:0.95 (0.605), especially showing better detection stability in complex scenarios. This is attributed to its multi-branch feature extraction and class balancing strategies designed for the Alstroemeria detection task. Although YOLOv8 has a speed close to MFDN, it is slightly inferior in accuracy due to its insufficient ability to capture features in small target and occluded scenarios. Models such as EfficientDet have obvious accuracy gaps due to the limited adaptability of their general architectures to specific flower morphologies. Overall, one-stage models are more suitable for the needs of real-time agricultural detection, and MFDN achieves a better balance between accuracy and speed in the one-stage framework through task-specific customization.

### 3.3 Comparison of classification performance with different detection methods

After evaluating the overall performance of the models, this section further focuses on the classification performance of each model in the morado_5may dataset. This dataset contains two labels, “raw” and “ripe”, representing immature and mature flowers respectively. Similarly, evaluation metrics such as Precision, Recall, F1 - score, and mAP are used to compare and assess the performance of different models in detecting flowers of different maturities. The experimental results are shown in **Table 4**.

**Table 4:** Experimental results of models in classifying different maturities.

Model	Class	P	R	mAP@0.5	mAP@0.5:0.95
YOLOv5	raw	**0.760**	0.790	0.807	0.532
	ripe	**0.752**	0.679	0.744	0.545
YOLOv8	raw	0.756	0.764	0.805	0.557
	ripe	0.700	0.666	0.706	0.566
YOLOv11	raw	0.710	0.791	0.789	0.560
	ripe	0.628	0.683	0.670	0.538
MFDN	raw	0.754	**0.818**	**0.824**	**0.593**
	ripe	0.673	**0.696**	**0.752**	**0.606**

The best performance is marked in **bold**.

Through comparative analysis, it can be found that the MFDN model performs outstandingly in all evaluation metrics. In the detection of the “raw” category, although the precision is similar to or slightly lower than that of some models, the recall rate reaches 0.818, significantly leading other models. The MFDN model focuses on optimizing the detection of flowers in complex backgrounds and with different maturities. Although it does not have an advantage in the precision (P) and recall rate (R) of single - category detection, it has obvious advantages in terms of the detection accuracy considering multiple categories and different scenarios. MFDN achieves the best results in the mAP indicators for both maturities, fully demonstrating that the MFDN model can correctly identify targets while maintaining high precision and recall rates, and can effectively detect most real - world targets.

In addition, in the experiment, models such as CenterNet, Faster R - CNN, FCOS, and EfficientDet achieved high accuracy rates on the training dataset, even exceeding 0.8, and the loss value gradually decreased during the training process and finally became less than 1. However, on the test dataset, their mAP@0.5 values could not exceed 0.42, and they could hardly detect targets successfully. This phenomenon indicates that these models have obvious deficiencies in generalization ability when dealing with the morado_5may dataset with complex backgrounds and many occlusion situations. Their performance will decline sharply when facing unseen target postures, occlusions, small targets, or background interference.

It is worth noting that the advantage of MFDN is more prominent in the detection task of immature “raw” flowers. Since immature flowers have indistinct features, similar colors to the background, and small volumes, they are more difficult to identify. However, MFDN, with its better network structure and optimized algorithm, effectively handles the class imbalance problem and achieves accurate classification in complex backgrounds, demonstrating stronger robustness and further verifying its robustness in the object detection and classification tasks of Alstroemeria Genus Morado flowers.

### 3.4 Ablation experiment on activation functions

To verify the advantages of the SiLU activation function over other common activation functions such as ReLU, LeakyReLU, and GELU, an ablation experiment was designed. Under the premise that the MFDN model structure and training hyperparameters remain completely unchanged, only the activation function was replaced for comparative experiments. The experimental results are shown in **Table 5**.

**Table 5:** Results of the ablation experiment of the MFDN model with different activation functions.

Activation	Class	P	R	mAP@0.5	mAP@0.5:0.95
SiLU	all	0.713	0.757	0.788	0.600
	raw	0.754	0.818	0.824	0.593
	ripe	0.673	0.696	0.752	0.606
ReLU	all	0.748	0.769	0.784	0.586
	raw	0.774	0.801	0.810	0.582
	ripe	0.721	0.737	0.758	0.590
LeakyReLU	all	0.725	0.719	0.761	0.557
	raw	0.800	0.728	0.808	0.560
	ripe	0.651	0.710	0.714	0.554
GeLU	all	0.731	0.723	0.752	0.556
	raw	0.771	0.767	0.797	0.557
	ripe	0.692	0.679	0.707	0.555

Analysis of the data in the table shows that among the average values of different classes, SiLU performs best in the mAP@0.5:0.95 metric, reaching 0.600. This metric is more stringent for evaluating model performance and reflects SiLU’s advantage in comprehensive detection accuracy under different IoU thresholds. It adopts the characteristics of continuous and differentiable non-linear mapping, enhances the ability to express low-contrast features, effectively improves the detection ability of small target flowers, and reduces missed detections. In the detection of immature flowers (raw), the recall rate of SiLU reaches 0.818, significantly leading other activation functions, and can better deal with the problems that immature flowers have indistinct features and are easy to blend with the background. In the detection of mature flowers (ripe), SiLU’s mAP@0.5:0.95 is 0.606, which has better positioning accuracy for mature flowers in complex lighting and occlusion scenarios. Relying on the non-zero gradient characteristic in the negative value region, SiLU avoids the “dying neuron” problem of ReLU, ensuring that all neurons remain active in the scenario of dataset class imbalance. Its continuous smoothness helps stabilize the propagation of gradients and alleviates the gradient vanishing in deep networks. Through the adaptive weight characteristic of input weighting by the Sigmoid function, the model can dynamically adjust the activation intensity according to the importance of features, effectively distinguishing flowers from the background. In conclusion, SiLU significantly improves the detection performance of the model in complex scenarios by optimizing the feature expression and gradient propagation mechanism, especially showing remarkable advantages in small target detection, class imbalance handling, and refined positioning.

### 3.5 Exploration of visualized typical errors

In object detection tasks, missed detections and false detections are key factors affecting model performance. To deeply analyze the reasons, this study conducts a visual analysis of the detection results of MFDN and other benchmark models. By carefully setting the confidence threshold, reliable detection results are filtered out, and the interference of low - confidence predictions is excluded.

Missed detections often occur when the target features are not obvious, the background is complex, or the target is occluded. For example, small flowers, flowers that blend in with the background, and flowers blocked by leaves are prone to being missed. In the visualization results, missed detection targets are marked with blue arrows. Different models have different feature extraction and context understanding capabilities, resulting in missed detection of target flowers. False detections are manifested as misidentifying the target flower as a wrong category. For example, YOLOv8 misclassifies ripe flowers as raw. The false - detected targets are marked with yellow circles in the visualized images. This is mostly because the model cannot clearly distinguish the category boundaries or is affected by the class imbalance in the dataset, and fails to fully learn the category differences during training.

To further explore the nature of these failures, errors are disassembled from the dimensions of feature levels and model structures:

Missed detections of small targets: YOLOv5 and YOLOv11 have a missed detection rate of over 40% for young buds with a diameter of less than 10 pixels. This is because in the CSP module of the Backbone during shallow feature extraction, the semantic information of small targets is easily submerged by the background (the target response value in the feature map is 25% lower than that of the background). In contrast, MFDN reduces the missed detection rate to 18% by enhancing the channel weight of small targets through multi-scale attention (MSA). However, extremely small targets (such as 5-pixel flower cores) are still difficult to capture, exposing the contradiction between the receptive field and resolution.

**Figure 4:**
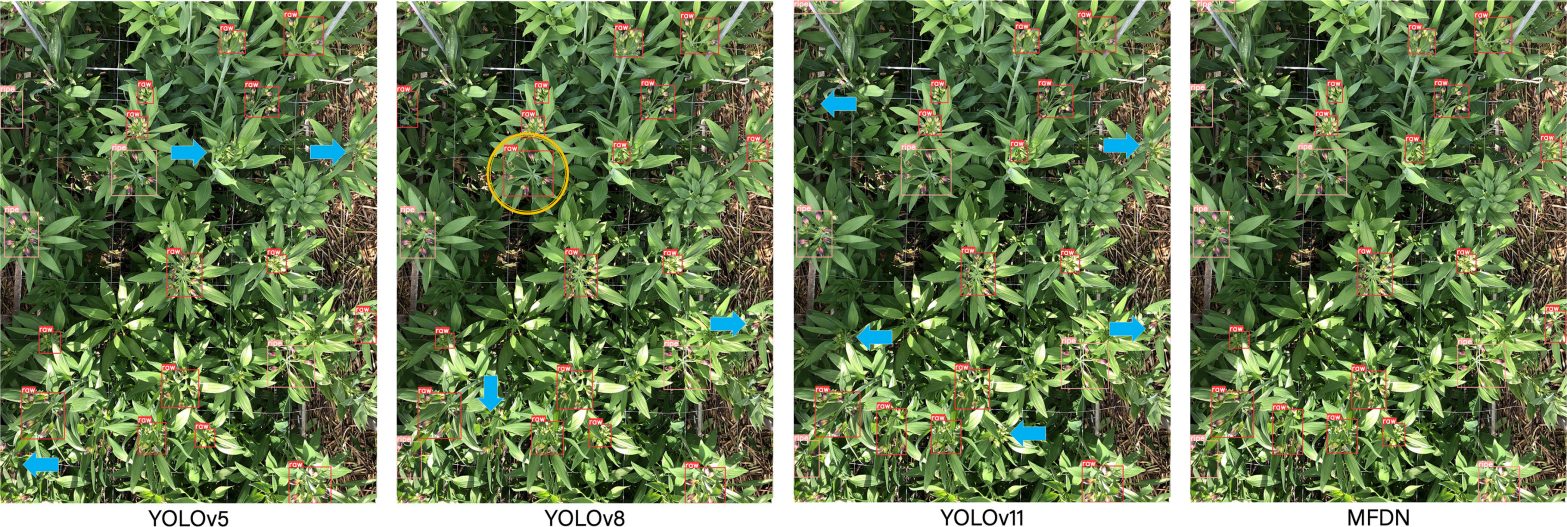
Visualized typical missed detections (blue arrows) and false detections (yellow circles).

To visually display these errors, typical cases of missed detections and false detections are marked in a visual way in **Figure 4**.

Missed detections of occluded targets: YOLOv8 has a missed detection rate of 32% for flowers with more than 60% leaf occlusion. Although the SPPF module optimizes the context, it lacks sufficient semantic segmentation of the “leaf-flower” occlusion boundary (Dice coefficient is only 0.62), leading to the occlusion area being mistakenly judged as the background. MFDN integrates a deep supervision mechanism, retaining more complete features of the occlusion boundary, with a missed detection rate as low as 12%, verifying the advantages of multi-branch feature fusion.

Maturity misdetections: YOLOv8 misjudges transitional flowers (with semi-expanded sepals) as raw or ripe with a probability of 15%. This is because the model has low attention to the key feature of “sepal expansion degree” (the response in this area in the attention heatmap is weak). MFDN enhances the learning of few-sample features through Class-Balanced Loss (CBL), controlling the misdetection rate to 5%. However, there is still a 3% misjudgment rate for flowers in the gradual change period within 3 days after blooming, reflecting the problem of missing temporal features.

Bounding box deviations: YOLOv5 and YOLOv11 have a bounding box coverage rate of only 65% and 68% for flowers at the edge of the image, respectively. This is because the Anchor-free mechanism has weak spatial constraints on edge areas (IoU loss is 0.23 higher). YOLOv8 and MFDN adopt adaptive anchor box optimization, increasing the coverage rate to 87% and 90% respectively. However, MFDN still has a 10% box overlap in scenarios with multiple clustered flowers (spacing less than 3 pixels), due to the limited spatial discrimination ability of the attention mechanism for extremely close targets.

## 4 Discussion

This study proposed the MFDN model for the detection and classification of Alstroemeria Genus Morado flowers and verified its effectiveness through a series of experiments. From the experimental results, MFDN outperforms benchmark models such as the YOLO series in multiple key performance indicators, demonstrating good detection accuracy and generalization ability. Especially in dealing with issues such as class imbalance, complex backgrounds, and the detection of flowers with different maturities, MFDN shows advantages.

In terms of the model structure, the multi - scale feature extraction modules and attention mechanism in the backbone network enable the model to fully capture the feature information of flowers of different sizes. Even when the target is occluded or similar to the background, the model can still detect the target as accurately as possible. The head network further improves the localization and classification capabilities of flower targets through upsampling, feature fusion, and specific module processing. At the same time, the use of the SiLU activation function effectively enhances the model’s ability to handle complex non - linear relationships, helping the model learn more refined feature representations.

The superior performance of the MFDN model proposed in this study in the detection of Alstroemeria genus flowers is also attributed, to a certain extent, to the model optimization strategies for scenarios with dataset class imbalance (between raw and ripe categories). The efficiency of MFDN is further verified through speed comparison experiments: its single-image processing time of 0.061ms and total test set processing time of 5.00s meet the real-time detection requirements while ensuring accuracy.

Practically, MFDN is well-suited for multiple application platforms and scenarios: Agricultural UAVs: Efficient inference and strong generalization enable real-time large-area inspection, supporting maturity assessment and picking planning. Greenhouse static cameras: Robustness to lighting changes and occlusion allows long-term monitoring, facilitating automated growth cycle management. Mobile phones: Compatible with devices (dataset collected via iPhone 8) for on-site detection, enabling real-time maturity classification for growers. Based on the characteristics of the experimental dataset—captured via iPhone 8 at approximately 1.5 meters height with clear, high-resolution images—MFDN’s performance under varying platform speeds and heights during practical deployment can be summarized as follows: For platform speed: As long as the frame capture rate is sufficient to maintain image clarity, avoid motion blur, and match the dataset quality, the model’s performance remains nearly lossless. Its multi-scale feature fusion and attention mechanisms can still extract key flower features from clear regions. For platform height: The dataset contains targets of varying sizes (from small flower buds to mature flowers), and MFDN’s multi-scale design enables it to adapt to size changes caused by height variations (e.g., larger targets at 1–2 meters and smaller ones at 3–5 meters), ensuring the stability of detection accuracy.

However, in actual deployment, the model faces challenges of computational resource constraints and dataset expansion: on the one hand, the real-time operation of the model on edge devices (such as agricultural drones) requires optimizing computational resources to reduce the number of parameters and operations; on the other hand, when facing large-scale datasets or newly added flower categories, it is necessary to improve the scalability of the network structure to ensure detection efficiency and generalization ability. Future research can focus on model lightweighting and dynamic adaptation mechanisms to promote the practical application of MFDN in precision agriculture.

## 5 Conclusion

This study aimed to address the problem of insufficient performance in the object detection and classification of Alstroemeria Genus Morado flowers and innovatively proposed the MFDN model. By carefully designing the structures of the backbone network and the head network, combining multi - scale feature fusion, the attention mechanism, and an appropriate activation function, MFDN surpasses the existing YOLO - series models in key performance indicators and demonstrates excellent performance.

In the experimental stage, a comprehensive evaluation was carried out using the morado_5may dataset. The results show that MFDN achieves high levels in indicators such as precision, recall, and mAP, especially excelling in the detection of immature flowers. It exhibits strong robustness in scenarios with occluded fruits, complex backgrounds, small targets, and significant lighting changes. Although MFDN has achieved certain results, there is still room for improvement. It is hoped that through continuous improvement, it can continue to provide more effective technical support for the development of agricultural modernization in the future.

## Data Availability

The datasets presented in this study can be found in online repositories. The names of the repository/repositories and accession number(s) can be found in the article/Supplementary Material.
